# Preoperative parathyroid harpoon localisation: a new technique helpful in reoperative patients with persistent hyperparathyroidism

**DOI:** 10.1308/003588413X13511609955896

**Published:** 2013-03

**Authors:** J Gómez-Ramírez, D Tagarro, JM Bravo, E Martín-Pérez, E Larrañaga

**Affiliations:** La Princesa University Hospital, Madrid,Spain

**Keywords:** Persistent hyperparathyroidism, Harpoon localisation, Reoperation

## Abstract

Surgery for persistent primary hyperparathyroidism remains a major challenge for surgeons and these reoperative procedures require an experienced parathyroid surgeon. The goal of reoperative surgery is to excise the abnormal parathyroid gland(s) and limit exploration to help minimise the potential complications. At least two positive and concordant localising studies should be available before reoperation because the technical difficulties in these cases make an exact localisation necessary before surgery. We describe the placement of a metallic harpoon under ultrasonography guidance as a safe, simple and inexpensive technique for localisation of the enlarged gland prior to conservative surgery.

The surgical management of primary hyperparathyroidism (pHPT) has evolved over the past two decades towards a more selective approach. It is well known that pHPT is mostly sporadic and is caused by a single adenoma in 85–95% of cases.^1^ A careful medical history and precise preoperative identification of the enlarged gland by parathyroid technetium sestamibi scintigraphy and neck ultrasonography allows selecting patients for minimally invasive parathyroidectomy.

Persistent pHPT is a challenging problem for surgeons.^2^ The technical difficulties posed by the scar tissue and distorted anatomy in the reoperative neck make exact localisation necessary before surgery. Compared with the initial operations, reoperations for persistent or recurrent hyperparathyroidism are associated with higher complication rates. There are numerous benefits to focused parathyroidectomy for these patients. Preoperative localisation of parathyroid adenomas in patients with persistent hyperparathyroidism relies currently on a combination of ultrasonography, technetium sestamibi scintigraphy/single photon emission computed tomography (SPECT), magnetic resonance imaging and venous sampling of parathyroid hormone. No procedure is universally reliable, however, and in reoperations for missed parathyroid adenomas, development of an optimal preoperative localisation strategy becomes especially problematic. This paper describes a novel and minimally invasive method of parathyroid localisation.

## Case history

**Figure 1:**
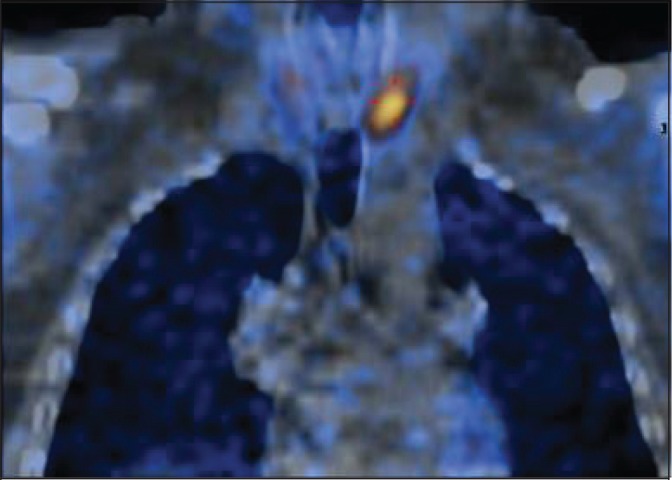
The single photon emission computed tomography showed an enlarged gland at the left side of the neck, just behind the inferior pole of the thyroid.

**Figure 2:**
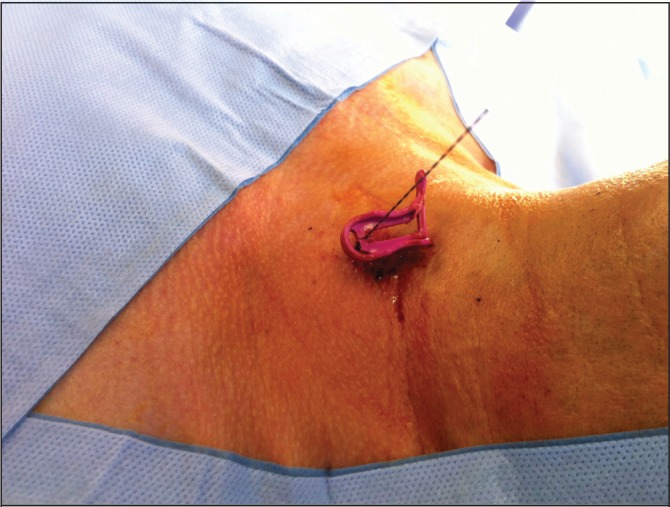
A metallic harpoon device is introduced before surgery in the parathyroid gland.

**Figure 3:**
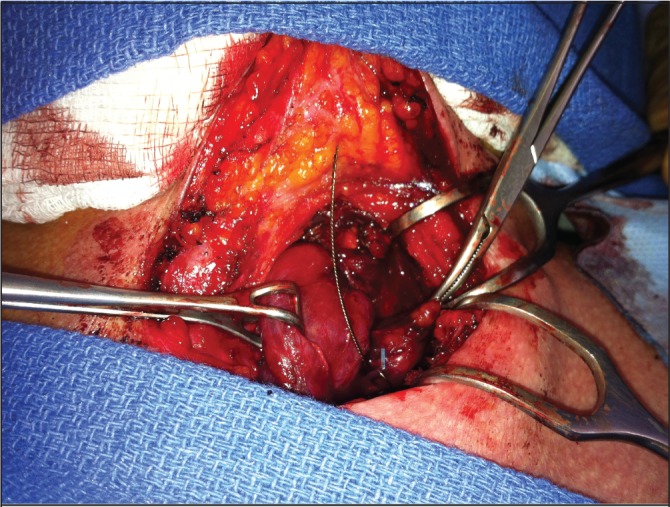
The harpoon was followed with dissection until the lesion was identified.

**Figure 4:**
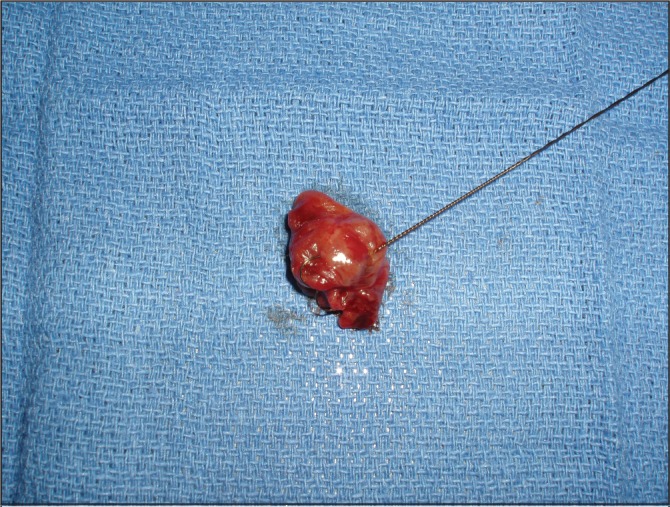
The parathyroid adenoma is resected.

A 57-year-old man with a medical history of hypertension and nephrolithiasis was referred for an elective parathyroidectomy because of symptomatic persistent pHPT after a previous parathyroid exploration at an outside facility. Ultrasonography and SPECT were performed to localise the adenoma. Both showed an enlarged gland at the left side of the neck, just behind of the inferior pole of the thyroid (Fig 1). Before surgery, a metallic harpoon device was introduced under ultrasonography guidance to the appropriate position in the suspect parathyroid gland (Fig 2). The skin incision was made to include the point of entry of the guidewire and the harpoon was followed with dissection until the lesion was identified (Fig 3). The mass containing the hook wire was subsequently dissected and excised (Fig 4). Serum calcium and parathyroid hormone levels decreased postoperatively and histopathology confirmed the diagnosis of a parathyroid adenoma.

## Discussion

Reoperative parathyroid surgery may be required in patients who have persistent pHPT after an unsuccessful operation and remains a major difficulty for surgeons. The goal of reoperative surgery is to excise the abnormal parathyroid gland and limit exploration to minimise the potential complications. These reoperative procedures require an experienced parathyroid surgeon armed with intraoperative adjuncts to locate the offending parathyroid gland(s) and remove them while minimising collateral injury.^3^

## Conclusions

In these patients who undergo persistent hyperparathyroidism, the placement of a metallic harpoon under ultrasonography guidance is a safe, simple and inexpensive technique for localisation of the enlarged gland prior to conservative surgery. Although harpoon placement is not indicated in all patients with persistent pHPT and although it does not solve the problem of adenomas that are difficult to find in patients where the preoperative imaging is negative, this technique can be helpful in high risk patients with prior neck surgery if the lesion is easy enough to find on ultrasonography. In these patients, reoperation can be very difficult because of the scar tissue and distorted anatomy, making exact localisation necessary before surgery.
